# Community-based models of care for management of type 2 diabetes mellitus among non-pregnant adults in sub-Saharan Africa: A scoping review

**DOI:** 10.1371/journal.pone.0278353

**Published:** 2023-11-15

**Authors:** Emmanuel Firima, Lucia Gonzalez, Fabiola Ursprung, Elena Robinson, Jacqueline Huber, Jennifer M. Belus, Fabian Raeber, Ravi Gupta, Gibrilla F. Deen, Alain Amstutz, Bailah Leigh, Maja Weisser, Niklaus Daniel Labhardt

**Affiliations:** 1 Division of Clinical Epidemiology, University Hospital Basel, Basel, Switzerland; 2 Faculty of Medicine, University of Basel, Basel, Switzerland; 3 Clinical Research Unit, Swiss Tropical and Public Health Institute, Basel, Switzerland; 4 Faculty of Biomedical Sciences, Universita della Svizzera Italiana, Lugano, Switzerland; 5 SolidarMed, Swiss Organisation for Health in Africa, Maseru, Lesotho; 6 College of Medicine and Allied Health Sciences, University of Sierra Leone, Freetown, Sierra Leone; 7 Department of Internal Medicine, Connaught Hospital, Freetown, Sierra Leone; 8 Division of Infectious Diseases and Hospital Epidemiology, University Hospital Basel, Basel, Switzerland; 9 Chronic Diseases Clinic, Ifakara Health Institute, Ifakara, Tanzania; The University of Sydney, AUSTRALIA

## Abstract

**Introduction:**

The prevalence of type 2 diabetes mellitus (T2DM) and associated morbidity and mortality are increasing in sub-Saharan Africa (SSA). To facilitate access to quality care and improve treatment outcomes, there is a need for innovative community care models and optimized use of non-physician healthcare workers bringing diagnosis and care closer to patients’ homes.

**Aim:**

We aimed to describe with a scoping review different models of community-based care for non-pregnant adults with T2DM in SSA, and to synthesize the outcomes in terms of engagement in care, blood sugar control, acceptability, and end-organ damage. We further aimed to critically appraise the different models of care and compare community-based to facility-based care if data were available.

**Methods:**

We searched Medline, Embase, Cumulative Index to Nursing and Allied Health Literature (CINAHL) and Scopus, supplemented with backward and forward citation searches. We included cohort studies, randomized trials and case-control studies that reported on non-pregnant individuals diagnosed with T2DM in SSA, who received a substantial part of care in the community. Only studies which reported at least one of our outcomes of interest were included. A narrative analysis was done, and comparisons made between community-based and facility-based models, where within-study comparison was reported.

**Results:**

We retrieved 5,335 unique studies, four of which met our inclusion criteria. Most studies were excluded because interventions were facility-based; community care interventions described in the studies were only add-on features of a primarily facility-based care; and studies did not report outcomes of interest. The included studies reported on a total of 383 individuals with T2DM. Three different community care models were identified. 1) A community-initiated model where diagnosis, treatment and monitoring occurred primarily in the community. This model reported a higher linkage and engagement in care at 9 months compared to the corresponding facility model, but only slight reductions of average blood glucose levels at six months compared to baseline. 2) A facility-originated community model where after treatment initiation, a substantial part of follow-up was offered at community level. Two studies reported such a model of care, both had as core component home-delivery of medication. Acceptability of this approach was high. But neither study found improved T2DM control when compared to facility care 3) An eHealth model with high acceptability scores for both patients and care providers, and an absolute 1.76% reduction in average HbA1c levels at two months compared to baseline. There were no reported outcomes on end-organ damage. All four studies were rated as being at high risk for bias.

**Conclusion:**

Evidence on models of care for persons with T2DM in SSA where a substantial part of care is shifted to the community is scant. Whereas available literature indicates high acceptability of community-based care, we found no conclusive data on their effectiveness in controlling blood sugar and preventing complications. Evidence from larger scale studies, ideally randomized trials with clinically relevant endpoints is needed before roll-out of community-based T2DM care can be recommended in SSA.

## Introduction

Diabetes mellitus (DM) is one of the most common chronic diseases worldwide [1–4], and majority of patients present with type 2 DM (T2DM) [5, 6]. In sub-Saharan Africa (SSA), about 24 million people currently live with DM and this number is expected to more than double by 2045 [4, 7]. Untreated or poorly treated T2DM leads to end-organ complications including retinopathy, peripheral sensory neuropathy, nephropathy, and cerebrovascular accidents [8]. These complications are among the most important contributors to mortality and disability [5, 9–11]. Traditionally, management of patients with diabetes in SSA is carried out in health facilities [12], or with occasional community linkages as ‘add-on’ service [13]. Patients within this care model go through clinics that are often congested, distant from their homes or working places, and must wait long hours to access care [12]. High direct and indirect costs are majorly borne by patients as out-of-pocket payments [14]. Poor access to care as a result of traditional models of care and rising costs have led to under-diagnosis, under-treatment and consequently poor health outcomes for people living with T2DM in these settings [12, 14].

The Sustainable Development Goals set to reduce by 2030 the burden of non-communicable diseases, and achieve universal health coverage [15]. To meet these ambitious targets, health models that increase access to care especially in low-resource regions like SSA must be developed, validated, and scaled-up [16]. Such models will need to reduce cost, be acceptable, feasible, safe and effective. Although primary care centers could potentially fill this gap, decentralizing T2DM care to primary care centers has been sub-optimal, with unsatisfactory outcomes [17, 18]. As such, health models that merely introduce community linkages as ‘add-ons’ to primarily facility-based care models will not be enough. Instead, community-based models that effectively reduce the frequency of patient contact with health facilities to reduce cost to patients, and cost and workload at the clinics will be needed [16, 19, 20].

Community-based care refers to interventions delivered outside of formal health facilities [13, 20]. It includes the services of professionals in residential and community settings in support of self-care, home-care, long-term-care and treatment [20]. A systematic review assessing studies in different low- and middle-income settings showed the usefulness of community-based programs to improve outcomes in immunization programmes, uptake of breast feeding and adherence to tuberculosis treatment [21]. Another systematic review and meta-analysis on the effect of community-based programs on T2DM prevention in low- and middle-income countries [1] revealed that such programs had positive outcomes on patients at risk of T2DM. A recent scoping review indicates substantial potential of community-based care models for arterial hypertension in SSA [22]. Moving care of uncomplicated cases and low-risk groups to the community level and to non-physician health workers, has advantages including fewer clinic visits, not having to travel long distances, not waiting in queues, and freeing up medical services in the facility for complicated cases, and high-risk groups, such as pregnant women with gestational diabetes requiring more specialized care. Ideally such models result in efficient and good quality care to both the groups receiving care in the community and those receiving care in the facility [23].

Currently, there is a lack of evidence on the effectiveness of community-based care for management of T2DM in the SSA region. We conducted a scoping review to map currently existing models of T2DM community-based care among non-pregnant adults in SSA; synthesize evidence on clinical outcomes of those care models in terms of engagement in care, blood sugar control, end-organ damage, as well as acceptability to both patients and care providers; and to compare the performance of the community-based models of care to facility-based care, if reported.

## Materials and methods

We conducted this scoping review using the framework initially developed by Arskey and O’Malley, and further refined by Levac et al. and the Joanna Briggs Institute [24–26]. Ethics approval was not needed as all data for this review were retrieved from already published studies. The study protocol with detailed description of our method has been published [27]. Briefly, we searched Medline, Embase, Cumulative Index to Nursing and Allied Health Literature (CINAHL) and Scopus on 23^rd^ May 2021 and 15^th^ October 2021 using the following keywords: “community-based care”, “type 2 diabetes” and “sub-Saharan Africa”. Our final search was conducted on the 24^th^ October 2022 to update the first search. (The search string is available on S1 Table in S1 File). If screened articles described study protocols that were topically relevant, first authors of those articles were contacted for any initial data on their studies. Forward and backward citation searches were carried out on articles that were included after full text screening.

We included studies carried out in SSA which reported community models of care where the majority of care was delivered outside of, and reduced frequency of patient contact with, traditional health facilities. In studies where care delivered in the community did not reduce contact with health facilities, such community-based care was considered ‘add on’ care, and the studies excluded. We only included studies that reported at least one of the following outcomes: engagement in care, blood glucose indices, T2DM complications, or acceptability of care to patients and providers. See Table 1 for PICO framework.

**Table 1:** PICO framework.

Criteria	Determinants
Population	Adult persons with non-gestational type 2 diabetes mellitus in sub-Saharan Africa
Intervention	Community-based care delivery
Comparison	Facility-based care (where available)
Outcome	Acceptability to patients and/or healthcare workers, Fasting blood glucose, Random blood glucose, glycated haemoglobin (HbA1c), engagement in care, development of T2DM-related complications

Since definition of engagement in care differed between studies, we adopted the definition used in the respective study. T2DM complications included development of retinopathy, neuropathy, nephropathy or diabetic foot syndrome. Acceptability of care was the uptake and utilization of the models of care by the patients or healthcare providers. As acceptability of care is variously defined [28], we adopted scales used by authors of the respective study. There was no restriction on language. We excluded studies on pregnant women and patients below 18 years of age. The rationale for excluding pregnant women was that gestational diabetes constitutes a separate entity with specific care and treatment requirements that differ from T2DM. We included studies that were prospective or retrospective cohorts, randomised controlled trials, non-randomised controlled trials, and quasi-randomised controlled trials. See S2 and S3 Tables in S1 File.

All search results identified using respective search strings for Medline, Embase, Cumulative Index to Nursing and Allied Health Literature (CINAHL) and Scopus were imported into EndNote™ and de-duplicated. Initially, two reviewers (EF and FU) independently screened all abstracts, applying the pre-defined eligibility criteria. Abstracts were excluded if they did not meet our inclusion criteria; or included for full text screening if they either met our inclusion criteria or if eligibility could not be determined immediately. Afterwards, full texts of all included studies were retrieved. Reviewers (EF and FU) independently screened the full texts for inclusion. Any disagreements were resolved by discussions between EF, LGF and NDL. Studies which were initially included but excluded during screening of the full text were specifically labelled as such in a table of excluded studies including the reason for exclusion.

A data extraction tool was created in Word™ and designed to collect information on author, year of publication, study design, location of study, duration of follow-up, type of community-based care model, health provider cadre, special trainings administered to providers and outcomes assessed (see S4 Table in S1 File). Where applicable, outcomes in a comparator arm (facility-based care) were also extracted. Data extraction was done independently and in duplicate by EF and FU. Discrepancies were discussed and resolved in consultation with a third person (NDL).

A textual narrative synthesis approach was used for analysis and synthesis [29]. In a first step, we identified and classified the model(s) evaluated in each study. For this, we used the framework on primary care-based models of NCD care in SSA by Kane et al. [30]. This framework classifies models of care according to origin or source of included patients; key activities undertaken within the care model; key cadre of participating staff; additional staff preparation for model; integration with other care; follow up and evaluation plan; and outcome. Afterwards, the components of each model were summarized. Findings are presented using tables and narrative reporting.

The Newscastle-Ottawa scale [31, 32] was used to assess the quality of the cohort studies. The scale grades selection, comparability, and outcome domains to an overall maximum score of 9. Although thresholds are not validated, we adopted the approach proposed in a recent review where scores of less than 6 are considered to be of high risk of bias [33]. We assessed the randomized controlled trial using the Cochrane Collaboration’s tool for assessing risk of bias in randomized controlled trials [34]. We assessed bias in the randomization process, deviation from intended intervention, completeness of outcome data for each main outcome, bias in the measurement of outcome and bias in the selection of the reported result [35].

## Results

**Fig 1:**
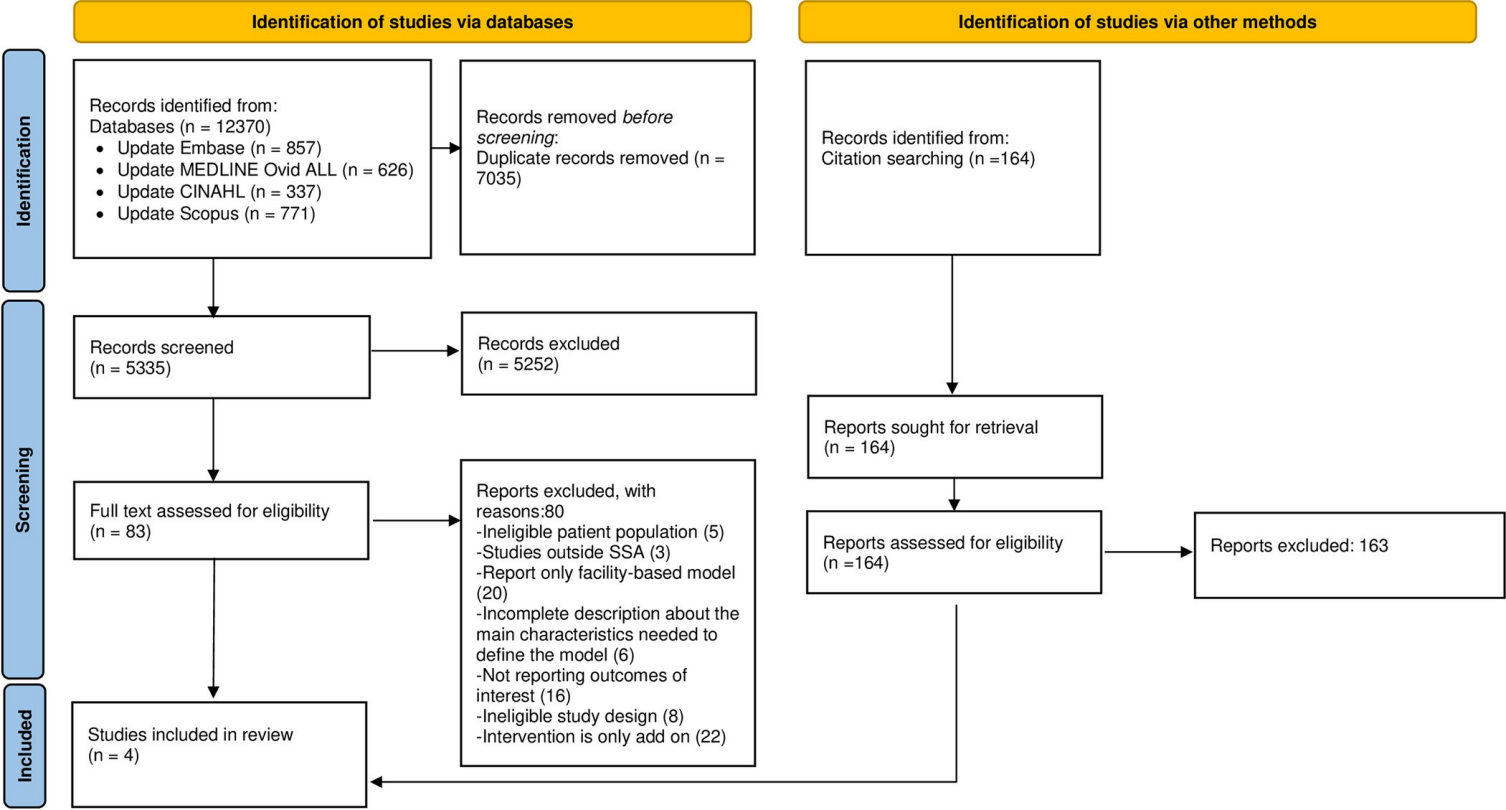
Flow chart showing result of database search and screening of primary articles.

After de-duplication, our database search yielded 5,335 records. Additionally, we retrieved 164 articles from backward and forward citation search. We assessed 83 full text articles out of which 4 articles met our inclusion criteria and are included in this scoping review. See PRISMA [36] diagram in Fig 1. Reasons for exclusion were: non-eligible patient population (pre-diabetes, pregnant women, type 1 diabetes); primarily facility-based model for delivering treatment with community-based model as add-on; incomplete description about the main characteristics needed to define the model; not reporting relevant outcomes; non-eligible study design. Numbers of studies excluded for each reason are presented on the flow chart in Fig 1.

### Study characteristics

Characteristics of the four studies including design and intervention are summarized on Table 2. The first study was a mixed-method study conducted in an urban setting in South Africa [37]. The quantitative aspect of this study was a prospective cohort with a non-randomly selected control group. The second study was an observational cohort study with a historical control group conducted in a rural setting in Kenya [38]. The third study was a randomized pilot trial without formal comparison between the two groups conducted in an urban area in the Democratic Republic of Congo [39]. The fourth study was a retrospective cohort study with a matched control group, conducted in an urban area in South Africa [21]. Overall, the four studies report on *N* = 383 T2DM patients within the community care models.

**Table 2:** Summary of main study characteristics.

	Author and publication year	Study design	Country	Setting	Participants (eligibility criteria)	Sample size (*n*)	Study period	Community care intervention
**1**	David et al. 2022 [37]	Mixed method	South Africa	Urban	• Existing diagnosis of T2DM • Prescribed metformin and/or glimepiride	• 331 in community model • 130 in facility model	16 months	**Home delivery of medication**: • CHW delivery of pre-packaged medication • Assessment of symptoms of COVID-19 • Assessment of other complaints • Needs-based referral to primary health facility.
**2**	Pastakia et al. 2017 [38]	Prospective cohort with historic control group	Kenya	Rural	For screening: all consenting individuals. For cohort: all diagnosed with T2DM	876 screened, 10 with T2DM	6 to 12 months	**BIGPIC:** • Community screening, • Linkage to peer/microfinance group. • monthly microfinance meetings • T2DM treatment in community
**3**	Takenga et al. 2014 [39]	Randomized pilot trial	Democratic Republic of Congo	Urban	T2DM patients between the age of 35 to 75	40 (20 in intervention arm)	Not reported	**Mobil Diab System**: • eHealth solution available as mobile app or web-based. • Enables self-monitoring of blood glucose by patients. • Remote follow-up by care providers. • Telephone support
**4**	Ndou et al. 2013 [21]	Retrospective cohort study with matched control group	South Africa	Urban	Stable patients with diabetes.	56 community model (22 with T2DM); 168 facility care (42 with T2DM)	Not reported	**Kgatelopele programme:** • Monthly packaging of medications by pharmacist. •Monthly home visits, where CHW brings the medication to the patient’s home. •6-monthly clinic visits by patients to be examined by physician.

BIGPIC = Impact of Bridging Income Generation with Group Integrated Care; T2DM = type 2 diabetes mellitus; CHW = community health workers

### Models of care

Based on the framework by Kane et al. [30], we identified three models of care: community-initiated model; eHealth model; and facility-originated community model (see Table 3).

**Table 3:** Main components of the community care interventions described presented by care model categories.

Community care intervention	Source of patients	Location of care	Key activities undertaken	Cadre of staff used	Additional staff preparation for model delivery	Integrated care	Outcome measured
**Community-initiated model**							
BIGPIC	Community-based screening, enrolment of individuals diagnosed with diabetes	Households and places used for gatherings like religious centers, schools, markets and shops	Medical prescription and drug distribution, lifestyle change support	Community care workers^a^, local volunteers, social workers, pharmacists, clinical officers, and physicians	Not reported	Health education with agro-business adviceeconomic stability through loans within microfinance groupsDemand for care creation through incentives • aHT co-management	Blood glucose, HbA1c Linkage to care Engagement in care
**eHealth model**							
Mobil Diab System	Known T2DM patients, source of recruitment not reported	Long distance support using eHealth tools e. g specially deployed app	Blood glucose monitoring by patient and reported through eHealth tool	Physician^b^	Not reported	None	HbA1c, Acceptability to patient Acceptability to healthcare provider
**Facility-originated community model**							
Home delivery of medication	Primary health centers	Patient’s household	Medication prescription and distribution	community health worker^a^		Co-management with aHT	HbA1c, Acceptability to patient
Kgatelopele programme	Primary health centers	Patient’s household	Medication prescription and distribution to patient’s home. Health promotion, education, adherence counselling.	Physician, pharmacist, community health worker^a^	14-week training for CHW on home care, adherence counselling and health promotion	Co-management with aHT	Blood glucose

BIGPIC = Impact of Bridging Income Generation with Group Integrated Care; T2DM = type 2 diabetes mellitus; aHT = arterial hypertension

^a^Main staff cadre providing services.

^b^Self-care driven, with physician supervision

The Impact of Bridging Income Generation with Group Integrated Care (BIGPIC) [38] intervention is a community-initiated model, which was developed in rural Kenya and focused on diabetes and hypertension care. The intervention included community-based screening and diagnosis; linkage to a peer group and a microfinance group; integration of health education and business counselling; incentives to generate demand for care; and care provision in the community. The study reported on blood glucose, linkage to and engagement in care. It compared linkage to, and engagement in care with a historical facility-based comparison group. In BIGPIC, primarily non-physician clinicians screened, diagnosed, and treated patients in the community either at or near the patient’s home. An important additional component of BIGPIC was integration of diabetes care with hypertension care and economic empowerment.

The Mobile Diab System [39], an e-health model used for patients already diagnosed and treated for T2DM, was implemented in two urban areas of the Democratic Republic of Congo. This model aimed to improve access to care, reduce frequency of clinic visits and increase patient’s involvement. With this model, patients were able to self-monitor their health, for example track exercises, drug intake and blood glucose measurement. These data were then sent via a portal to the patient’s physician who reviewed the information. Feedback, including therapy adjustments, instructions or recommendations were sent back to patients through the mobile system. Central to this model were long-term medication prescription and distribution, self-care, self-monitoring and clinician follow-up via eHealth platforms. The study reported on blood glucose levels, HbA1c as well as acceptability of the care model to both patients and care providers. The model empowered patients, ensured information flow between providers and patients, and reduced the frequency of clinic visits.

Two studies fell into facility-originated model, the Kgatelopele programme and the Home Delivery of Medication (HDM) intervention. The Kgatelopele programme [21] sought to improve care for people living with diabetes and hypertension by providing home care through community health workers. The overall aim was to improve acceptability, accessibility, and affordability of care.

Activities undertaken were monthly packaging of medications by pharmacists, monthly home visits by community health workers who then brought the packed medications to patients, and six-monthly clinic visits. Although the main staff cadre involved was the community health worker, this programme included specialized care from pharmacists and physicians at the clinic. Community health workers also provided social support geared towards improved patient literacy about their condition, adherence to medication and clinic visits. Patients were recruited at the clinic and outcomes were retrospectively compared to a matched cohort from the same clinic who did not enroll in the community programme. The Kgatelopele programme was similar to the community-initiated model BIGPIC, with the main exception that it was a facility-originated community model where known and stable patients were transferred from clinic- to community-based care. As in the community-initiated model, care and medication were delivered by non-physician health workers. Like BIGPIC, the Kgatelopele programme integrated T2DM management with care for hypertension.

Home Delivery of Medication (HDM) [37] intervention was developed due to difficulties faced in health facilities during the Coronavirus disease (COVID-19) pandemic. The aim of the intervention was to determine if patient follow-up in the community with HDM improved blood glucose control and acceptability by patients. Community health workers delivered pre-packaged medications to eligible patients. They also performed various evaluations including assessment for COVID-19 symptoms and other complaints with referral to primary health facilities if needed.

### Reported outcomes and comparisons to facility-based model of care

The BIGPIC study from Kenya reported on linkage to care, engagement in care, blood glucose and HbA1c. The Mobil Diab pilot trial from the Democratic Republic of Congo reported on acceptability by patients and providers, blood glucose values and HbA1c, and the Kgatelopele study reported blood glucose values only. The HDM intervention reported on HbA1c and acceptability by patients. There were no reported outcomes on end-organ damage. See Table 4.

**Table 4:** Summary of outcomes and comparisons where relevant.

Author and publication year	Study period/timing of outcome	Model	Acceptability		Blood sugar level*		Linkage and engagement in care	
			Model outcome	Conventional care outcome	Model outcome	Conventional care outcome	Model outcome	Conventional care outcome
Pastakia et al. 2017 [38]	6 to 12 months	Community-initiated		-	FBS: 160.4mg/dl at baseline; 153.2mg/dl after 6 months. HbA1c: 10.8% at baseline; 10.0% after 6 months	-	100% 10/10 with T2DM linked to care 70% engagement in care at 9 months	31% linkage to care
Takenga et al. 2014 [39]	2 months	eHealth	For patients: Mean score of 8.65 on a scale of 1 to 10. For medical staff: Mean score of 8.75	-	HbA1c: 8.65% at baseline; 6.89% at 2 months	HbA1c: 8.59% at baseline; 8.6% at 2 months	-	-
Ndou et al. 2013 [21]	NR	Facility-originated community model	-	-	FBS 3.6–5.8 mmol/L: 9.1%	FBS 3.6–5.8mmol/L: 26.1%	-	-
David et al. 2022 [37]	16 months	Facility-originated community model	100% acceptability of model	-	HbA1c: 9.3% at baseline; 9.5% at follow-up 28% of patients with controlled^#^ DM at baseline; 27% of patients with controlled DM at follow-up	HbA1c: 9.6% at baseline; 10.1% at follow-up 27% of patients with controlled DM at baseline; 22% of patients with controlled DM at follow-up		

NR = not reported; FBS = fasting blood sugar; HbA1c = glycated haemoglobin. * Reported FBS and HbA1c values are averages.

^#^ Controlled DM for the intervention was defined as HbA1c < 7.5%

### Engagement in care

Linkage to care, which was defined as return to subsequent group meeting following positive screening for T2DM, was 100% for BIGPIC’s community model compared to 31% in the historic facility-based comparison group. At 9 months, seven (70%) of the 10 T2DM patients were still in care. Linkage to care, and engagement in care were reported only in BIGPIC’s community care model.

### Blood glucose control

In the Kgatelopele program, blood glucose control was defined as a fasting blood glucose of between 3.6 to 5.8 mmol/L among people with T2DM. Applying this definition, 2/22 (9%) and 11/42 (26%) achieved control in the community and the facility care group, respectively. In BIGPIC’s community-initiated model, average fasting blood glucose in the 10 patients with T2DM was 160.4mg/dL (8.9 mmol/L) at baseline and reduced to 153.2mg/dL (8.5 mmol/L) after 6 months representing a non-significant reduction. Average HbA1c decreased from 10.8% at baseline to 10.0% after 6 months. Blood glucose indices were not reported for the historical control group. Within the Mobil Diab system’s eHealth model, baseline average HbA1c was 8.65% with a 1.76 percentage points reduction to 6.89% at 2 months. This model was compared to conventional care where at 2 months average HbA1c remained unchanged (baseline 8.59%, 2-months 8.6%). For the HDM intervention, average baseline HbA1c values were 9.3% and 9.6% and increased to 9.5% and 10.1% in the community- and the facility-based model, respectively.

### Acceptability

Acceptability was reported by both HDM intervention and Mobil Diab system’s eHealth model. For the HDM intervention, implemented during the COVID-19 pandemic, patients’ acceptability was assessed by questionnaires. All interviewed patients preferred HDM because it was convenient, safe, resolved their transport difficulties, and reduced their fear of getting infected in the facility. Within Mobil Diab system’s eHealth model, acceptability to patients and acceptability to healthcare providers were described on a 10-point scale based on responses to two questions, each directed to patients and to healthcare providers. The questions explored whether patients and healthcare providers would wish to continue using the system, and whether they would recommend the system to others. The average score to both questions was then taken as the acceptability score. The scores were 8.65 and 8.75 out of 10 for patients and healthcare workers respectively. These results, according to the authors, reflected an overall positive acceptance of the system by the patients and health workers. The authors also stated that one reason for patient acceptance of the model was the motivation to reach target blood glucose levels since patients could easily access the database which showed carbohydrate contents of the meals they were taking. For medical practitioners, the system was suitable because of the possibility to supervise more patients simultaneously and remotely. The major concern for both patients and health workers were internet cost and time required to get familiar with both mobile and web applications. Suggestions to improve acceptability and performance of the eHealth platform included updating the drug and food list in the apps to include more locally available items, making available more trainings about use of the system, introducing glucose measuring device kit, and creating within the model an avenue for sporting activities for patients.

### Quality of evidence

HDM intervention, BIGPIC and Kgatelopele cohort studies scored below 6, thus classified as being of high risk of bias. The low scores were mainly due to a lack of comparators (BIGPIC) or non-randomly selected comparators (Kgatelopele programme, HDM intervention). See S5 Table in S1 File. The included randomized pilot trial had overall high risk of bias. The randomization domain was found to be of high risk of bias. There were some concerns related to deviation from intended intervention, measurement of the outcome and selection of the reported result. See S6 Table in S1 File. Further, reporting of the trial did not follow standards for randomized controlled trials [40].

## Discussion

We conducted a scoping review to describe published data from cohort and intervention studies that assessed community-based models of care for people living with T2DM in SSA where a relevant part of care was provided outside the facility. To our knowledge, this is the first review that addresses this question. Our literature search strategy yielded four eligible articles out of 5,335 distinct records. The four studies were very heterogeneous in design, type of care model described, and outcomes reported. Quality of evidence provided by the studies as well as the relatively low number of T2DM patients enrolled in the four community models of care make it impossible to conclude on the effectiveness of community-based T2DM care in SSA. As such, this scoping review’s main finding is that there is a considerable evidence gap regarding community-based T2DM care in SSA, and larger scale, well designed studies are needed. Such future studies may build on the experience reported in the four studies included in this review.

The Kgatelopele’s programme, a facility initiated community model of care, reported only on blood glucose levels. After treatment initiation at the facility, significant aspects of patient care were moved to the community for 22 selected patients among whom only two achieved blood sugar control. In contrast, 46 of the 168 (26%) remaining in facility-based care achieved glycemic control. In BIGPIC’s community-initiated model, with 10 T2DM patients, there was only a very modest reduction in fasting blood glucose and HbA1c at six months follow-up. Similarly, the HDM Intervention found no clear indication of better T2DM control in the cohort receiving home delivered medication. These results differ from other reports from LMICs [1] which showed significant reductions in fasting blood glucose and HbA1c measurements in favor of community-based models care where the community-aspect was an add-on to and did not replace standard facility care.

In one of the studies assessed in our full text review but excluded from the scoping review [41], the intervention supported self-monitoring of blood glucose and relied on six approaches: identification of high risk patients by clinicians and enrollment in the intervention by community health workers; sending patients home with a glucometer and cell phone access; weekly follow-up via phone calls for blood glucose results; glucose results and medication dose summaries generated for clinician to review. Although utilizing an eHealth model for follow-up, the study was not included in this review as majority of participants had type 1 diabetes mellitus. However, average HbA1c was reduced from 13.3% at baseline to 9.1% at 6 months.

Unsurprisingly, BIGPIC’s community model increased linkage to care, performing better than conventional care. Although there was a 70% engagement in care at 9 months in this model, comparison data was not reported for conventional care. However, the high linkage to care with the model is similar to other studies [42]. Adopting community care approaches has resulted in better care engagement in various diseases [43]. Our finding thus suggests that adopting community models of care has the potential to improve linkage and engagement in care for diabetes management in SSA. The success seen in linkage to care and engagement in care reported within BIGPIC’s community model possibly resulted from their peer-based approach, in line with reports from other studies [44, 45]. Further, the micro-finance component which made funds available and promoted income-generating activities likely contributed to increased linkage and engagement in care.

The HDM intervention, and the Mobile Diab system’s eHealth model reported high acceptability to both patients and health workers. Although there was no comparison of ‘acceptability’ to conventional care, several other studies report high acceptability of eHealth by end-users [46, 47]. A recent study evaluating the implementation of home delivery of medication for various illness during the COVID-19 pandemic found that patients and providers preferred the continuance of this approach, with overall improvements in patient adherence to medication [48]. The eHealth model observed a clinically relevant reduction of HbA1c [49]. In a systematic review conducted to determine the effectiveness of telemedicine in the delivery of diabetes care in low- and middle-income countries [11], telemedicine yielded significant reductions in HbA1c, with interventions via telephone and short message service yielding the highest treatment effects [11].

A major gap uncovered in this scoping review is the paucity of studies on community models of care that reduce the frequency of patient contact with health facilities to reduce cost to patients, and cost and workload at clinics. Additionally, while our review suggests that community models could improve T2DM care, included studies were generally of a low quality. Thus, there is a need for more research, preferably randomized controlled trials, to assess the effectiveness of community care. Incorporating aspects on economic and social determinants of the outcome of community-based T2DM management would provide further insights. Some of the community interventions in this review integrated T2DM care with arterial hypertension and economic activities. A recent systematic review [50] suggests that integrated care in sub-Saharan Africa is feasible. Although the systematic review focused on integration of HIV and diabetes, community care for T2DM should also seek to integrate care for other chronic conditions.

Our scoping review has several limitations. First, as we did not target grey literature, we may have missed some models of care. Second, design, patient recruitment and outcome definition and evaluation differed across included studies making comparison of outcomes impossible. For instance, acceptability was assessed differently between the two studies that reported the outcome. Third, the overall number of T2DM patients enrolled in the four community-based models of care was relatively low. Fourth, lack of comparison groups in some of the included studies made it difficult to fully interpret the findings in the studies. Fifth, as two of the retrieved four studies were conducted in South Africa, their findings may not be generalizable to Sub-Saharan Africa. Finally, all studies had substantial flaws in design and/or reporting making conclusions on effectiveness of community-based care for T2DM in SSA impossible.

## Conclusion

Although community-based care for patients with T2DM in SSA may be a promising approach to improve access to diagnosis and care, current evidence on such models is very limited. We identified only four studies reporting on models of care in SSA where a substantial part of the management was moved from the facility to the community. In total these four studies report on 383 patients with T2DM enrolled in one of these care models. The studies hint at opportunities and challenges community-based T2DM care may provide. However, larger scale studies, ideally randomized, with mid- to long-term outcomes on key-indicators such as engagement in care, HbA1c, occurrence of diabetes complications and cost to health systems and patients are needed before a roll-out of community-based care for T2DM patients in SSA can be recommended.

## Supporting information

S1 File Containing S1 to S6 Tables, and S1 Appendix. (DOCX)

## References

[1] Shirinzadeh M, Afshin-Pour B, Angeles R, Gaber J, Agarwal G. The effect of community-based programs on diabetes prevention in low- and middle-income countries: a systematic review and meta-analysis. Globalization and Health. 2019;15(1):10. doi: 10.1186/s12992-019-0451-4 30709362 PMC6359819

[2] Ogurtsova K, da Rocha Fernandes JD, Huang Y, Linnenkamp U, Guariguata L, Cho NH, et al. IDF Diabetes Atlas: Global estimates for the prevalence of diabetes for 2015 and 2040. Diabetes Res Clin Pract. 2017;128:40–50. doi: 10.1016/j.diabres.2017.03.024 28437734

[3] Saeedi P, Petersohn I, Salpea P, Malanda B, Karuranga S, Unwin N, et al. Global and regional diabetes prevalence estimates for 2019 and projections for 2030 and 2045: Results from the International Diabetes Federation Diabetes Atlas, 9(th) edition. Diabetes Res Clin Pract. 2019;157:107843. doi: 10.1016/j.diabres.2019.107843 31518657

[4] Sun H, Saeedi P, Karuranga S, Pinkepank M, Ogurtsova K, Duncan BB, et al. IDF Diabetes Atlas: Global, regional and country-level diabetes prevalence estimates for 2021 and projections for 2045. Diabetes research and clinical practice. 2022;183:109119. doi: 10.1016/j.diabres.2021.109119 34879977 PMC11057359

[5] Diagnosis and classification of diabetes mellitus. Diabetes Care. 2010;33 Suppl 1(Suppl 1):S62–9. 20042775 10.2337/dc10-S062 PMC2797383

[6] Kerner W, Brückel J. Definition, classification and diagnosis of diabetes mellitus. Exp Clin Endocrinol Diabetes. 2014;122(7):384–6. doi: 10.1055/s-0034-1366278 25014088

[7] Cho NH, Shaw JE, Karuranga S, Huang Y, da Rocha Fernandes JD, Ohlrogge AW, et al. IDF Diabetes Atlas: Global estimates of diabetes prevalence for 2017 and projections for 2045. Diabetes Res Clin Pract. 2018;138:271–81. doi: 10.1016/j.diabres.2018.02.023 29496507

[8] Li S, Wang J, Zhang B, Li X, Liu Y. Diabetes Mellitus and Cause-Specific Mortality: A Population-Based Study. Diabetes Metab J. 2019;43(3):319–41. doi: 10.4093/dmj.2018.0060 31210036 PMC6581547

[9] Harding JL, Pavkov ME, Magliano DJ, Shaw JE, Gregg EW. Global trends in diabetes complications: a review of current evidence. Diabetologia. 2019;62(1):3–16. doi: 10.1007/s00125-018-4711-2 30171279

[10] Nisar MU, Asad A, Waqas A, Ali N, Nisar A, Qayyum MA, et al. Association of Diabetic Neuropathy with Duration of Type 2 Diabetes and Glycemic Control. Cureus. 2015;7(8):e302. doi: 10.7759/cureus.302 26430576 PMC4571902

[11] Correia JC, Meraj H, Teoh SH, Waqas A, Ahmad M, Lapão LV, et al. Telemedicine to deliver diabetes care in low- and middle-income countries: a systematic review and meta-analysis. Bull World Health Organ. 2021;99(3):209–19B. doi: 10.2471/BLT.19.250068 33716343 PMC7941107

[12] Sharp A, Riches N, Mims A, Ntshalintshali S, McConalogue D, Southworth P, et al. Decentralising NCD management in rural southern Africa: evaluation of a pilot implementation study. BMC Public Health. 2020;20(1):44–. doi: 10.1186/s12889-019-7994-4 31931762 PMC6956511

[13] Lankester T. 3Community-based health care: Setting the scene. In: Lankester T, Grills N, Lankester T, Grills NJ, editors. Setting up Community Health and Development Programmes in Low and Middle Income Settings: Oxford University Press; 2019. p. 0.

[14] Mutyambizi C, Pavlova M, Chola L, Hongoro C, Groot W. Cost of diabetes mellitus in Africa: a systematic review of existing literature. Globalization and Health. 2018;14(1):3. 29338746 10.1186/s12992-017-0318-5 PMC5771003

[15] Organization WH. World health statistics 2016: monitoring health for the SDGs sustainable development goals: World Health Organization; 2016.

[16] Bertram MY, Sweeny K, Lauer JA, Chisholm D, Sheehan P, Rasmussen B, et al. Investing in non-communicable diseases: an estimation of the return on investment for prevention and treatment services. Lancet. 2018;391(10134):2071–8. doi: 10.1016/S0140-6736(18)30665-2 29627159

[17] Pfaff C, Malamula G, Kamowatimwa G, Theu J, Allain TJ, Amberbir A, et al. Decentralising diabetes care from hospitals to primary health care centres in Malawi. Malawi Med J. 2021;33(3):159–68. doi: 10.4314/mmj.v33i3.3 35233273 PMC8843181

[18] Mulugeta TK, Kassa DH. Readiness of the primary health care units and associated factors for the management of hypertension and type II diabetes mellitus in Sidama, Ethiopia. PeerJ. 2022;10:e13797. doi: 10.7717/peerj.13797 36042860 PMC9420406

[19] WHO. Global action plan for the prevention and control of noncommunicable diseases 2013–2020. World Heal Organ. 2013.

[20] Kielland Aanesen HA, Borras J. eHealth: The future service model for home and community health care. 2013 7th IEEE International Conference on Digital Ecosystems and Technologies (DEST) 2013. p. 172–7.

[21] Ndou T, van Zyl G, Hlahane S, Goudge J. A rapid assessment of a community health worker pilot programme to improve the management of hypertension and diabetes in Emfuleni sub-district of Gauteng Province, South Africa. Glob Health Action. 2013;6:19228. doi: 10.3402/gha.v6i0.19228 23364086 PMC3556684

[22] Fernández LG, Firima E, Robinson E, Ursprung F, Huber J, Amstutz A, et al. Community-based care models for arterial hypertension management in non-pregnant adults in sub-Saharan Africa: a literature scoping review and framework for designing chronic services. BMC Public Health. 2022;22(1):1126. doi: 10.1186/s12889-022-13467-4 35658850 PMC9167524

[23] Organization WH. Community-based health care, including outreach and campaigns, in the context of the COVID-19 pandemic: interim guidance, May 2020. World Health Organization; 2020.

[24] Arksey H O’Malley L. Scoping studies: towards a methodological framework. International Journal of Social Research Methodology. 2005;8(1):19–32.

[25] Levac D, Colquhoun H, O’Brien KK. Scoping studies: advancing the methodology. Implementation Science. 2010;5(1):69. doi: 10.1186/1748-5908-5-69 20854677 PMC2954944

[26] Peters MDJ, Marnie C, Tricco AC, Pollock D, Munn Z, Alexander L, et al. Updated methodological guidance for the conduct of scoping reviews. JBI Evid Synth. 2020;18(10):2119–26. doi: 10.11124/JBIES-20-00167 33038124

[27] Firima E, Gonzalez L, Huber J, Belus JM, Raeber F, Gupta R, et al. Community-based models of care for management of type 2 diabetes mellitus among non-pregnant adults in sub-Saharan Africa: a scoping review protocol. F1000Research. 2021;10(535):535. doi: 10.12688/f1000research.52114.2 35387273 PMC8961197

[28] Nadal C, Sas C, Doherty G. Technology Acceptance in Mobile Health: Scoping Review of Definitions, Models, and Measurement. J Med Internet Res. 2020;22(7):e17256. doi: 10.2196/17256 32628122 PMC7381045

[29] Barnett-Page E, Thomas J. Methods for the synthesis of qualitative research: a critical review. BMC Med Res Methodol. 2009;9:59–. doi: 10.1186/1471-2288-9-59 19671152 PMC3224695

[30] Kane J, Landes M, Carroll C, Nolen A, Sodhi S. A systematic review of primary care models for non-communicable disease interventions in Sub-Saharan Africa. BMC Fam Pract. 2017;18(1):46–. doi: 10.1186/s12875-017-0613-5 28330453 PMC5363051

[31] Gierisch J, Beadles C, Shapiro A, McDuffie J, Cunningham N, Bradford D. NEWCASTLE-OTTAWA SCALE CODING MANUAL FOR COHORT STUDIES [Internet]. Health Disparities in Quality Indicators of Healthcare Among Adults with Mental Illness [Internet]. Department of Veterans Affairs (US); 2014 [cited 2020 Sep 19]. 26065051

[32] Hartling L, Hamm M, Milne A. Decision rules for application of the Newcastle-Ottawa Scale. Agency for Healthcare, United States. 2012.

[33] Luchini C, Stubbs B, Solmi M, Veronese N. Assessing the quality of studies in meta-analyses: Advantages and limitations of the Newcastle Ottawa Scale. World J Meta-Anal. 2017;5(4):80–4.

[34] Sterne JA, Savović J, Page MJ, Elbers RG, Blencowe NS, Boutron I, et al. RoB 2: a revised tool for assessing risk of bias in randomised trials. bmj. 2019;366. doi: 10.1136/bmj.l4898 31462531

[35] Higgins JP, Savović J, Page MJ, Elbers RG, Sterne JA. Assessing risk of bias in a randomized trial. Cochrane handbook for systematic reviews of interventions. 2019:205–28.

[36] Page MJ, McKenzie JE, Bossuyt PM, Boutron I, Hoffmann TC, Mulrow CD, et al. The PRISMA 2020 statement: an updated guideline for reporting systematic reviews. BMJ. 2021;372:n71. doi: 10.1136/bmj.n71 33782057 PMC8005924

[37] David NJ, Bresick G, Moodaley N, Von Pressentin KB. Measuring the impact of community-based interventions on type 2 diabetes control during the COVID-19 pandemic in Cape Town—A mixed methods study. S Afr Fam Pract (2004). 2022;64(1):e1–e9. doi: 10.4102/safp.v64i1.5558 36073102 PMC9452916

[38] Pastakia SD, Manyara SM, Vedanthan R, Kamano JH, Menya D, Andama B, et al. Impact of Bridging Income Generation with Group Integrated Care (BIGPIC) on Hypertension and Diabetes in Rural Western Kenya. J Gen Intern Med. 2017;32(5):540–8. doi: 10.1007/s11606-016-3918-5 27921256 PMC5400758

[39] Takenga C, Berndt RD, Musongya O, Kitero J, Katoke R, Molo K, et al. An ICT-Based Diabetes Management System Tested for Health Care Delivery in the African Context. Int J Telemed Appl. 2014;2014:437307. doi: 10.1155/2014/437307 25136358 PMC4127241

[40] Antes G. The new CONSORT statement. British Medical Journal Publishing Group; 2010. doi: 10.1136/bmj.c1432 20332507

[41] Pastakia SD, Cheng SY, Kirui NK, Kamano JH. Dynamics, Impact, and Feasibility of Self-Monitoring of Blood Glucose in the Rural, Resource-Constrained Setting of Western Kenya. Clin Diabetes. 2015;33(3):136–43. doi: 10.2337/diaclin.33.3.136 26203206 PMC4503944

[42] Musicha C, Crampin AC, Kayuni N, Koole O, Amberbir A, Mwagomba B, et al. Accessing clinical services and retention in care following screening for hypertension and diabetes among Malawian adults: an urban/rural comparison. J Hypertens. 2016;34(11):2172–9. doi: 10.1097/HJH.0000000000001070 27552644 PMC5790170

[43] Rich ML, Miller AC, Niyigena P, Franke MF, Niyonzima JB, Socci A, et al. Excellent clinical outcomes and high retention in care among adults in a community-based HIV treatment program in rural Rwanda. J Acquir Immune Defic Syndr. 2012;59(3):e35–42. doi: 10.1097/QAI.0b013e31824476c4 22156912

[44] Park PH, Wambui CK, Atieno S, Egger JR, Misoi L, Nyabundi JS, et al. Improving Diabetes Management and Cardiovascular Risk Factors Through Peer-Led Self-management Support Groups in Western Kenya. Diabetes Care. 2015;38(8):e110–1. doi: 10.2337/dc15-0353 26207058 PMC4512136

[45] Khabala KB, Edwards JK, Baruani B, Sirengo M, Musembi P, Kosgei RJ, et al. Medication Adherence Clubs: a potential solution to managing large numbers of stable patients with multiple chronic diseases in informal settlements. Trop Med Int Health. 2015;20(10):1265–70. doi: 10.1111/tmi.12539 25962952 PMC4744994

[46] Rahimi B, Nadri H, Lotfnezhad Afshar H, Timpka T. A Systematic Review of the Technology Acceptance Model in Health Informatics. Appl Clin Inform. 2018;9(3):604–34. doi: 10.1055/s-0038-1668091 30112741 PMC6094026

[47] Alshahrani A, Stewart D, MacLure K. A systematic review of the adoption and acceptance of eHealth in Saudi Arabia: Views of multiple stakeholders. Int J Med Inform. 2019;128:7–17. doi: 10.1016/j.ijmedinf.2019.05.007 31160014

[48] Mash RJ, Schouw D, Daviaud E, Besada D, Roman D. Evaluating the implementation of home delivery of medication by community health workers during the COVID-19 pandemic in Cape Town, South Africa: a convergent mixed methods study. BMC Health Services Research. 2022;22(1):98. doi: 10.1186/s12913-022-07464-x 35073888 PMC8784590

[49] Lameijer A, Fokkert M, Edens M, Slingerland R, Bilo H, van Dijk P. Determinants of HbA1c reduction with FreeStyle Libre flash glucose monitoring (FLARE-NL 5). Journal of clinical & translational endocrinology. 2020;22:100237. doi: 10.1016/j.jcte.2020.100237 33102135 PMC7578738

[50] Harrison SR, Jordan AM. Chronic disease care integration into primary care services in sub-Saharan Africa: a ’best fit’ framework synthesis and new conceptual model. Fam Med Community Health. 2022;10(3). doi: 10.1136/fmch-2022-001703 36162864 PMC9516220

